# History of previous surgery is associated with higher risk of revision after primary total knee arthroplasty: a cohort study from the Geneva Arthroplasty Registry

**DOI:** 10.1080/17453674.2021.1970322

**Published:** 2021-08-25

**Authors:** Hermes H Miozzari, Christophe Barea, Didier Hannouche, Anne Lübbeke

**Affiliations:** Division of Orthopaedics and Trauma Surgery, Geneva University Hospitals, University of Geneva, Faculty of Medicine, Geneva, Switzerland

## Abstract

Background and purpose — Prior to primary total knee arthroplasty (pTKA), 6–34% of patients have undergone surgical procedure(s) of their knee. We investigated whether history of previous surgeries influences the risk of revision of pTKA, the risk according to the type of previous surgery, and how previous surgery influences specific causes of revision and the time of revision.

Patients and methods — This is a prospective cohort study from the Geneva Arthroplasty Registry. All pTKA between 2000 and 2016 were included and followed until December 31, 2019. Outcomes were risk of revision, evaluated using Kaplan–Meier survival and Cox and competing risks regression, the specific causes, and time of revision.

Results — Of 3,945 pTKA included (mean age 71 years, 68% women), 21% had a history of previous surgery, with 8.3% revisions vs. 4.3%, at 3–20 years’ follow-up (mean 8.6). 5- and 10-year cumulative failure by previous surgery (yes vs. no) were 6.6% (95% CI 5.1–8.5) vs. 3.3% (CI 2.7–4.0), and 8.4% (CI 6.6–10.6) vs. 4.5% (CI 3.8–5.4). Baseline differences explained only part of the higher risk (adjusted HR 1.5, CI 1.1–2.1). The risk of failure was higher for all causes of revision considered. Patients in the previous surgery group had a higher risk of an early revision.

Interpretation — A history of previous surgery adversely affected the outcome with a 1.5 times higher cumulative risk of all-cause revision over the course of up to 20 years after index surgery. The increased risk was seen for all causes of revision and was highest in the first years.

The proportion of patients with a history of previous knee surgery before pTKA is documented in most but not all European national and a few local arthroplasty registries (Lübbeke et al. 2018), varying from 6% in Finland to 34% in Switzerland (Table 1). If previous surgery seems to affect both the age and interval for the need for pTKA, with patients undergoing arthroplasty at a significantly younger age (Brophy et al. 2014), it remains unclear whether this plays a substantial role in its outcome. According to recent data, 10-year pTKA survival rate seems not to be affected by arthroscopy (Viste et al. 2017), while the opposite was observed (87% vs. 98%) in an older study (Piedade et al. 2009). Data from the Norwegian (Badawy et al. 2015) knee arthroplasty registry did not show differences in survival rates in patients undergoing pTKA after high tibial osteotomy (HTO), while publications from the Swedish (Robertsson and W-Dahl 2015) and New Zealand (Pearse et al. 2012) registries showed a higher risk of revision. To our knowledge, there is only 1 study considering history of any given previous knee surgery (Lim et al. 2016), showing no difference in terms of revision.

**Table 1.** History of previous surgery in publicly available national joint registry reports and present study (GAR)

Country	Registry	Period	Published	History of previous surgery (%)
Belgium	ORTHOpride	2014–2018	10/2019	(29)
Finland	FAR	2014–2020	2020	4,300/70,288 (6)
Germany	EPRD	2010–2018	10/2019	(8)
Italy	RIAP	2006–2017	02/2018	(12)
Netherlands	LROI	2014–2018	Online	(31)
Norway	NAR	1994–2018	06/2019	1,449/6,905 (24)
Portugal	PAR	2009–2013		(7)
Sweden	SKAR	1975–2019	01/2020	(18)
Switzerland	SIRIS	2012–2018	2019	(34)
New Zealand	NZJR	1999–2018	12/2019	15,376/110,079 (14)
Geneva/Switzerland	GAR	1998–2019	–	(21)

Source: ISAR (International Society of Arthroplasty Registries (https://www.isarhome.org/members).

ORTHOpride, Belgian National Arthroplasty Register (https://www.ehealth.fgov.be/file/view/AXDOTDE0mTlaOSp4Nmeq?filename=Orthopride_Annual_Report_2018.pdf)

FAR, Finnish Arthroplasty Registry (https://www.thl.fi/far/#data/cphd)

EPRD, Endoprothesenregister Deutschland (https://www.eprd.de/fileadmin/user_upload/Dateien/Publikationen/Berichte/EPRD_Jahresbericht_2019_2.0.pdf)

RIAP, Italian Arthroplasty Registry (http://riap.iss.it/riap/en/activities/reports/2020/05/13/report-2018-english-addendum/)

LROI, Dutch Arthroplasty Register (https://www.lroi-rapportage.nl/media/pdf/PDF%20Online%20LROI%20annual%20report%202019.pdf)

NAR, Norwegian Arthroplasty Register (http://nrlweb.ihelse.net/Rapporter/Rapport2020.pdf)

PAR, Portuguese Arthroplasty Registry (http://www.rpa.spot.pt/getdoc/c3d0a244-c056-4949-a50b-07d0fdeac2b9/RPA-Report-2013.aspx)

SKAR, Swedish Knee Arthroplasty Register (http://myknee.se/pdf/SVK_2019_1.0_Eng.pdf)

SIRIS, Swiss National Joint Registry (https://www.siris-implant.ch/fr/Downloads&category=16)

NZJR, New Zealand Joint Registry (https://nzoa.org.nz/system/files/DH8328_NZJR_2019_Report_v4_7Nov19.pdf)

We compared revision rates up to 20 years after pTKA in a prospective cohort with and without previous surgeries. Our specific questions were: (i) Does history of previous surgeries influence the risk of revision of pTKA? (ii) What is the risk of revision according to the type of previous surgery? (iii) How does previous surgery influence specific causes of revision and the time of revision?  

## Patients and methods

### Study design and setting

We performed a prospective cohort study based on the local arthroplasty registry (Geneva Arthroplasty Registry, GAR). Since 1998, all patients undergoing partial, primary total, and revision knee arthroplasties are prospectively enrolled in the GAR, which was approved by the local ethics committee and is a member of the International Society of Arthroplasty Registries (ISAR).

### Participants/study subjects

All consecutive patients who underwent elective pTKA for any indication between January 2000 and December 2016 at the Geneva University Hospitals were included and followed up until December 31, 2019. The minimum follow-up was 3 years. The exposure of interest was the presence of previous surgery (yes/no). The outcome of interest was all-cause revision after pTKA.

### Surgery

The vast majority of the patients (99%) underwent pTKA by a standard medial parapatellar approach, with mechanical alignment, mostly with a postero-stabilized (PS) design. Patellar resurfacing has been performed in 68% of cases. Routine component fixation was by antibiotic-loaded cement (Palacos R + G, Heraeus Medical GmbH, Wehrheim, Germany). Preoperative single-shot antibiotic prophylaxis was done with either a second-generation cephalosporin or vancomycin, in the case of known allergy or bacterial resistance. Since 2017, the dosage has been doubled for patients with either BMI ≥ 35 or bodyweight ≥ 100 kg.

### Aftercare

Full weight-bearing with use of crutches was allowed from day 1, with full range of motion. Deep vein thrombosis prophylaxis was initiated immediately postoperatively and discontinued at 8 weeks. Physiotherapy was prescribed for at least 3 months.

### Variables, outcome measures, data sources, and bias

Data collection in the registry is done prospectively. Previous surgeries are documented in the electronic healthcare system and recorded in the registry in pre-specified categories as follows: arthroscopy, meniscectomy, meniscectomy external, meniscectomy internal, osteosynthesis, osteotomy, ligamentoplasty, others, or none. For ease, all meniscectomies were grouped in a single category as “meniscectomy.”

The following covariates were assessed: age, sex, BMI, ASA score, smoking status, diagnosis, patellar resurfacing, type of constraint (PS), type of tibial plateau (fixed bearing), year of surgery, and surgery duration.

All the information on baseline characteristics and operation, including previous surgery, are routinely recorded on specifically designed data collection forms by the operating surgeon at the time of surgery. Information on comorbidities (BMI, ASA score, smoking status) was collected from the anesthesiology chart. All data are routinely double-checked for completeness by the physician in charge (AL) to assure the quality of the registry.

The outcome all-cause revision was subdivided into revision due to aseptic loosening, infection, femoro-patellar problem, pain, arthrofibrosis, periprosthetic fracture, instability, and other causes. A revision is by definition any surgery with (partial) implant exchange or component extraction or resurfacing of the patella. Therefore, reoperation for manipulation under anesthesia, open/arthroscopic synovectomy, or any hardware removal did not account for the endpoint of the present study.

### Statistics

To assess the influence of previous surgeries on the risk of revision of pTKA, we first compared patient characteristics at baseline between the group who had undergone previous surgery and the group who had not. Information on previous surgery had been collected for all patients operated on during the inclusion period. Missing values on patient characteristics were: BMI in 17 cases (0.4%), ASA score in 4 (0.1%), and smoking status in 73 cases (1.9%). P-values were obtained using Student’s t-test for continuous variables and Pearson’s chi-square test for categorical variables.

To assess the risk of all-cause revision, cumulative failure analysis with all-cause revision as an endpoint was performed using the Kaplan–Meier method. Person-time at risk was determined as the length of the interval between date of surgery for the pTKA and the date of either revision for any reason, death, leaving the area of residency, or end of follow-up (December 31, 2019). We also performed both Cox regression and competing-risks analyses (with death as a competing event) and estimated unadjusted and adjusted hazard ratios (HR) with 95% confidence intervals (CI) (Fine and Gray 1999). Final adjustment variables included age, sex, ASA score, and year of surgery. We evaluated the primary outcome in all pTKAs and in only the first pTKA. Finally, we performed a subgroup analysis including only patients with pTKA for primary osteoarthritis.

To investigate whether the causes of revision were related to a history of previous surgery, the revision risk over the entire follow-up period was calculated for specific causes and stratified by previous surgery yes/no. Moreover, smoothed hazard estimates were obtained with their 95% CIs to evaluate whether the timing of the revisions differed between the 2 groups (Tanner and Wong 1983). Hazard estimates quantify the immediate risk, in this case of all-cause revision, attached to an individual known to be alive at time t.

The statistical analyses were performed using the statistical packages IBM SPSS statistics version 25 (IBM Corp, Armonk, NY, USA) and STATA version 15 (StataCorp, College Station, TX, USA).

### Ethics, funding, data sharing, and potential conflicts of interest

This study was approved by the local ethics committee (CCER Geneva, Switzerland). All the data used in this study is retrieved from the Geneva Arthroplasty Registry. The Division of Orthopaedic Surgery received financial institutional support from the “Fondation pour la recherche ostéo-articulaire” for the knee arthroplasty registry. The funding source had no role in the collection, analysis, or interpretation of the data, in the preparation of the manuscript, or its submission for publication. The Geneva Arthroplasty Registry obtains patient consent for data collection and protects access to the data. Patients gave consent to future sharing of data only upon request by other research institutions. Interested researchers may request access to data from DH. None of the authors report any conflict of interest.

## Results

### Study population (Table 2)

3,945 pTKAs (mean age 71 years, 68% women) were performed during the inclusion period, all enrolled in the registry and included in the final analysis. The mean follow-up time was 8.6 years (3–20 years). In the group of patients with previous surgery, the percentage of women (52% vs. 72%) was statistically significantly smaller, as well as the mean BMI (29 vs. 30), and the percentage of ASA ≥ 3 (19 vs. 28), whereas the proportion of ever smokers was significantly higher (35 vs. 24%). The percentage of patients with patellar resurfacing (69%) and fixed bearing (96%) was statistically significantly higher in the group without previous surgeries, whereas the amount of PS constraint was similar.

### Does history of previous surgeries influence the risk of revision of primary total knee arthroplasty?

**Figure 1.**
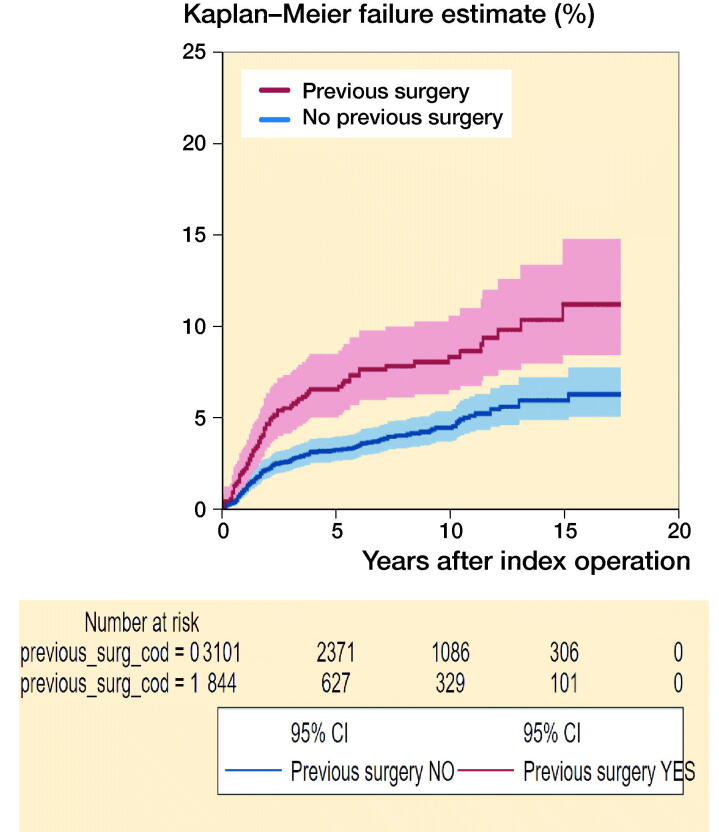
5- and 10-year cumulative failure for patients with and without history of previous surgery. 5-year cumulative failure (yes vs. no) was 6.6% (CI 5.1–8.5) vs. 3.3% (CI 2.7–4.0). 10-year cumulative failure was 8.4% (CI 6.6–11) vs. 4.5% (CI 3.8–5.4).

Of 3,945 pTKAs included in the study, 844 (21%) had a history of previous surgery, mostly meniscectomies (47%), followed by osteotomies (15%) and arthroscopies (15%) (see Table 2). At an average follow-up of 8.6 years, there were 204 revisions, 70 (8.3%) in patients with previous surgery and 134 (4.3%) in patients without. 5-year cumulative failure by previous surgery (yes vs. no) was 6.6% (CI 5.1–8.5) vs. 3.3% (CI 2.7–4.0). 10-year cumulative failure was 8.4% (CI 6.6–11) vs. 4.5% (CI 3.8–5.4) (Figure 1). Including all pTKAs, the unadjusted HR (Cox regression) for revision was 1.9 (CI 1.4–2.6) and 1.5 (CI 1.1–2.1) when adjusted for age, sex, ASA score, and year of surgery. Considering only the first pTKA implanted, the unadjusted HR was 2.0 (CI 1.5–2.8), and 1.6 (CI 1.2–2.2) when adjusted (Table 3). Corresponding estimates obtained with competing risks regression were similar.

**Table 2.** Baseline characteristics according to previous surgery yes/no (all primary TKAs). Values are count (%) unless otherwise specified

	Previous surgery		
	yes	no	
	n = 844	n = 3,101	
Factor	(21%)	(79%)	p-value
Women	439 (52)	2,233 (72)	< 0.001
Age at operation			
mean (SD)	67.3 (9.3)	72.5 (9.2)	< 0.001
BMI, mean (SD)^a^	29 (5.1)	30 (5.7)	< 0.001
BMI categories			
< 24.9	206 (24)	578 (19)	
25–29.9	321 (38)	1,106 (36)	
30–34.9	227 (27)	829 (27)	
≥ 35	89 (11)	572 (19)	
ASA score 3–4^b^	158 (19)	879 (28)	< 0.001
Ever smoker^c^	740 (35)	286 (24)	< 0.001
Bilateral primary TKA	88 (10)	625 (20)	< 0.001
Diagnosis primary OA	545 (65)	2,848 (92)	< 0.001
Previous knee surgery			
Arthroscopy	127 (15)	–	
Meniscectomy	400 (47)	–	
Osteosynthesis	75 (9)	–	
Osteotomy	129 (15)	–	
Ligamentoplasty	43 (5)	–	
Other	70 (8)	–	
Implant-related information			
Patellar resurfacing, yes	527 (62)	2,135 (69)	< 0.001
Posterior-stabilized, yes	677 (80)	2,496 (81)	0.9
Fixed-bearing, yes	780 (92)	2,974 (96)	< 0.001
Surgery duration, min (SD)	124 (31)	119 (28)	< 0.001

a BMI was missing in 17 cases (0.4%).

b ASA score was missing in 4 cases (0.1%).

c Smoking status was missing in 73 cases (1.9%).

**Table 3.** Hazard ratios (HR) for all-cause revision according to previous surgery yes/no

	Previous surgery (events/total number)		Cox regression		Competing-risks regression	
Included cases	yes	no	HR (CI)	aHR (CI) **^a^**	HR (CI)	aHR (CI) **^a^**
All TKAs	70/844	134/3,101	1.9 (1.4–2.6)	1.5 (1.1–2.1)	2.0 (1.5–2.6)	1.6 (1.2–2.1)
Only first TKA	67/756	109/2,476	2.0 (1.5–2.8)	1.6 (1.2–2.2)	2.1 (1.6–2.8)	1.6 (1.2–2.2)

a Adjusted for age, sex, ASA score and year of surgery using Cox regression and competing-risks regression

When restricting the inclusion to the diagnosis primary OA (n = 3,393) only, Cox regression estimates were only slightly higher than in the previous analyses. For all pTKA the unadjusted HR was 2.0 (CI 1.4–2.8) and the adjusted HR 1.7 (CI 1.2–2.4). Including only the first pTKA the unadjusted HR was 2.1 (CI 1.5–3.0) and the adjusted HR 1.7 (CI 1.2–2.5).

### What is the risk of revision according to the type of previous surgery?

The 5-year cumulative failure rates according to the type of previous surgery varied between 12% and 5.7% (Table 4).

**Table 4.** 5-year cumulative failure rates (and 95% CI) by type of previous surgery procedure (all TKAs)

Factor	Total number	Number of events	5-year cumulative failure rate (%)
No previous procedure	3,101	134	3.3 (2.7–4.0)
Previous procedure	844	70	6.6 (5.1–8.5)
Arthroscopy	127	15	7.9 (4.4–14)
Meniscectomy	400	29	5.7 (3.8–8.5)
Osteosynthesis	75	7	8.3 (3.8–18)
Osteotomy	129	7	4.1 (1.7–9.5)
Ligamentoplasty	43	3	7.1 (2.4–21)
Other	70	9	12 (6.1–22)

### How does previous surgery influence specific causes of revision and the time of revision?

**Figure 2.**
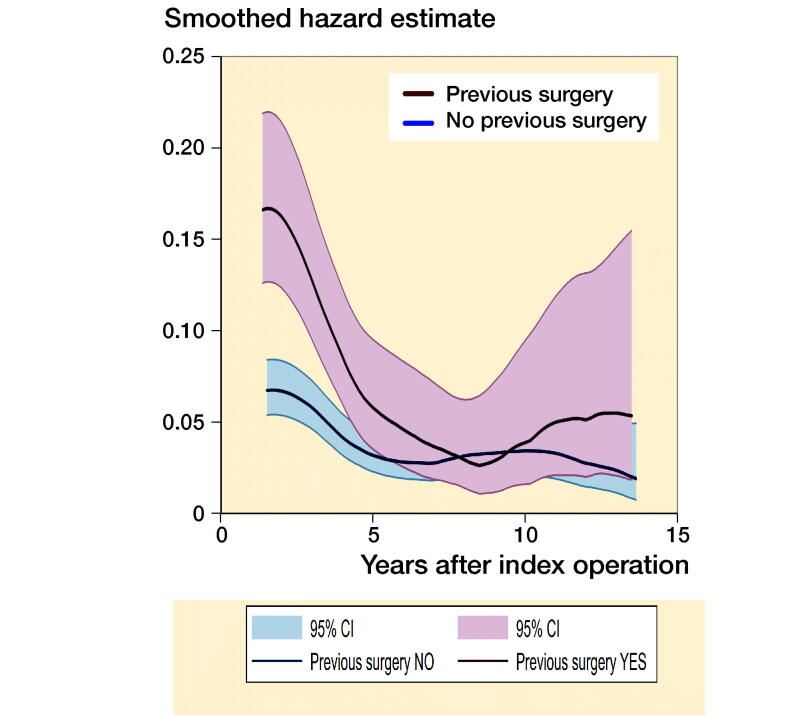
Smoothed hazard estimates of all-cause revision for patients with and without history of previous surgery. The risk of revision was substantially higher in the short term as evidenced by the distinct, non-overlapping confidence intervals.

The risk of revision in patients with vs. without previous surgery was almost twice as high for any specific causes, more often related to aseptic loosening (2.1% vs. 0.9%) or infection (1.9% vs. 1.2%) (Table 5). In terms of main procedures, all component revision was more common in patients without previous surgery whereas partial revision was mostly performed in patients with previous surgery (Table 6). The timing of revision differed between the 2 groups. The risk of revision was substantially higher in the short term as evidenced by the distinct, non-overlapping confidence intervals of the smoothed hazard estimates in Figure 2. The curves overlapped completely in the mid-term and partly in the long term. 

**Table 5.** Revision risk overall and for specific causes according to previous surgery, yes/no (all TKAs). Values are count (%)

	Previous surgery	
	yes	no
Revision cause	n = 844	n = 3,101
Aseptic loosening	18 (2.1)	29 (0.9)
Infection	16 (1.9)	36 (1.2)
Femoropatellar problem	9 (1.1)	11 (0.4)
Pain	8 (0.9)	17 (0.5)
Arthrofibrosis	6 (0.7)	8 (0.3)
Periprosthetic fracture	5 (0.6)	11 (0.4)
Instability	3 (0.4)	5 (0.2)
Other	5 (0.6)	17 (0.6)
All causes	70 (8.3)	134 (4.3)

**Table 6.** Categories of revision according to previous surgery, yes/no. Values are count (%)

	Previous surgery	
	yes	no
Revision	n = 70	n = 134
All component revision	21 (30)	57 (42)
Partial revision^a^	32 (46)	41 (31)
Poly exchange	15 (21)	26 (19)
Arthrodesis	0 (	2 (2)
TKA extraction	0 (	5 (4)
Other	2 (3)	3 (2)

a Partial revision: revision of either component (femoral, tibial, or patellar).

## Discussion

### Background and rationale

The proportion of patients with a history of previous surgery before pTKA reported in registries is highly variable (6–34%) (Table 1). Overestimation, due to multiple counting, underestimation, due to patient recall bias, incomplete chart fill or insufficient anamnesis, different current practices from one country to other, and different time periods included might all explain this variability. Nevertheless, it is not clear how a history of previous surgery influences the outcome after pTKA. In our study, patients in the group with previous surgery had primary arthroplasty at a younger age and showed a 1.5 times higher risk of subsequent revision. The risk did not substantially change when restricting the inclusion to primary OA. The difference in implant failure at 5 and 10 years was notable: about twice the risk at both time points (6.6% vs. 3.3 and 8.4% vs. 4.5%, respectively). The 2 groups’ baseline differences only partly explained the increased risk of revision, which was higher for any specific causes (from aseptic loosening to infection, etc.). The timing of revision differed between the 2 groups and was substantially higher in the short term in patients with pre-dating surgeries.

### Limitations

This study has several limitations. First, no analysis was carried out for single vs. multiple previous surgeries. The number of patients in whom it was clearly stated that they had more than 1 surgery at different points in time was too small to enable any further analysis (60 patients among 844). Other patients had more than 1 procedure noted but it was not possible to discriminate between different procedures for the same surgery or several surgeries at different time intervals. When more than 1 procedure was performed we considered the type of previous surgery that was first documented by the surgeon for calculation of failure by type of previous surgery. Second, we did not consider any surgery where the components were left untouched, such as manipulation under anesthesia, and open or arthroscopic synovectomy. Our results might underestimate the risk of reoperation. Nevertheless, our definition complies with the definition commonly used in registries, therefore allowing for comparison with the results of further studies. Third, in our analysis we could not differentiate between previous open and closed surgical procedures because our charts were not complete enough in this regard. This might be an important issue considering that past open surgical procedures are among the most influential risk factors for periprosthetic joint infection (Tan et al. 2018). Any past longitudinal scar included in the approach for pTKA might be unnoticed in the case of patient recall bias and if not reported in the charts at the time of arthroplasty. Finally, arthroscopy has been in use for diagnostic purposes, but it might well be that data entry for arthroscopy in the registry included a meniscectomy (external, internal) without mentioning this. Therefore, there is a risk of overestimation for arthroscopy and an underestimation for meniscectomy as a risk factor.

### Does history of previous surgeries influence the risk of revision of primary total knee arthroplasty?

The crude risk of all-cause revision after pTKA among those with a history of previous knee surgery was about twice as high as among those without (8.3 vs. 4.3%). Baseline differences in age, sex, ASA score, BMI, smoking status, patellar resurfacing, type of tibial plateau, and surgery duration partly explained the higher risk; it was, however, still 1.5 times greater after adjusting for the baseline imbalances. A subgroup analysis considering only the first pTKA implanted revealed similar results. Patients who had previous surgery were substantially younger, more often men, had fewer comorbidities including obesity, and were more often ever smokers. Similarly, Lim et al. (2016) highlighted that pTKA after previous surgery was performed at a younger age (61 vs. 66 years), even younger than ours (67 vs. 73 years). However, besides the age difference and a similar BMI of 27, we do not know if their groups differed in baseline characteristics compared with our cohort. Indeed, the etiology of osteoarthritis might be different. Our 2 groups were composed of different patient populations: younger, more active, vs. older, sicker patients, less active, and more obese. Work and sports-related accidents and the kind of work itself are more common risk factors in the first group than in the second where obesity prevails, for instance. The chance of having previous surgery such as ligament reconstruction, meniscal repair, osteotomies, or fracture repair is therefore higher in the first group.

### What is the risk of revision according to the type of previous surgery?

In this study, the risk of revision varied according to the type of previous surgery and it was lowest, with 4.1% (CI 1.7–9.5) 5-year cumulative failure rate in the case of previous osteotomy, and higher in the case of ligamentoplasty (7.1%), arthroscopy (7.9%), or previous osteosynthesis (8.3%). However, the confidence intervals around the estimates for the different types were large and overlapped considerably.

The kind of surgery might alter knee mechanics. Typically, previous osteotomies around the knee, or posttraumatic conditions, make TKA technically more challenging in terms of implant positioning and ligament balancing. But their effect on the revision risk is not evident. A study from the New Zealand Joint Registry (Pearse et al. 2012), showed a 3-fold increased risk of early revision in patients with a history of osteotomies around the knee, compared with pTKA without previous surgery, but the risk was not adjusted. In a more recent study from the Danish Knee Arthroplasty Registry (El-Galaly et al. 2018), 10-year survival of pTKA after HTO was inferior (91% vs. 94%), although this could be explained by lower age and male sex rather than the osteotomy (adjusted HR of 1.2 vs. a crude HR of 1.7). Another study from the same group reported an increased risk of early and mid-term revision of pTKA in the setting of OA after fractures around the knee (El-Galaly et al. 2017).

### How does previous surgery influence specific causes of revision and the time of revision?

The risk of revision after pTKA with previous surgery was about twice as high for any specific diagnosis, with aseptic loosening (2.1%) and infection (1.9%) being the most frequent (Table 5). The vast majority of patients in our cohort were homogeneously treated (80% PS, > 90% fixed bearing, 100% cemented pTKA), making implant-related factors unlikely to explain the difference in revision rates due to aseptic loosening. Both younger age (Khan et al. 2016) and a BMI over 35 (Abdel et al. 2015, Zingg et al. 2016) are known patient-related risk factors for revision, due to high activity levels and a higher mechanical load across the bone–cement interface, respectively. Nevertheless, our 2 groups were differently affected by these factors (those with previous surgery were younger, those without were more often obese), thus underlining the importance to control for previous surgery when comparing revision rates due to aseptic loosening.

The higher risk of infection encountered in patients with a history of previous surgery might be explained by an intrinsic risk due to previous interventions, as reported in a recent meta-analysis, with a RR of 3.0 (CI 1.5–5.9) (Kunutsor et al. 2016), especially with open surgical procedures (Tan et al. 2018), as well as a history of resolved septic arthritis following surgery or prolonged surgical time.

The amount of patellar resurfacing was lower in the group of patients with previous surgeries (62 vs. 69%) and partly explains the higher percentage of revision for femoro-patellar conflict in this group, as shown through the adjustment.

A fair number of patients were revised because of a “painful” pTKA, with no obvious cause of failure identified. Residual pain after pTKA is not unusual, but high patient expectations, years-long chronic pain situation, and social/economic pressure to resume work might all play a central role.

The higher prevalence of revision for instability in our series might be a consequence of post-traumatic conditions, consistent with a recent study from the Danish Knee Arthroplasty Register (El-Galaly et al. 2017). Concerning the timing of revision, their results are in line with our findings, with substantially more short-term revisions in the previous surgery group and no difference in the mid-term. In the long term, there might be a higher number of revisions in those with previous surgery. However, confidence intervals were overlapping.

## Conclusions

In this large cohort, 21% of patients undergoing pTKA had a history of previous surgery. The difference in implant failure at 5 and 10 years was notable, and the 2 groups’ baseline differences only partly explained the increased risk of revision. It is important to advise patients that their knee history adversely influences the outcome of pTKA, with a 1.5 times higher risk of revision, especially in the short term.

It would be of interest to know if data from other registries, including those with less frequent previous surgeries, supports our results. Future studies should analyze whether 1 vs. multiple surgeries prior to pTKA influences the survival differently and should focus on what causes of revision are related to a specific previous surgery, in an attempt to understand why this is and change our practice.
